# Linear Growth and Final Height in People With Type 1 Diabetes: A Study From Karachi, Pakistan

**DOI:** 10.7759/cureus.22397

**Published:** 2022-02-20

**Authors:** Wajid Shaikh, Musarrat Riaz, Saima Askari, Abdul Basit

**Affiliations:** 1 Baqai Institute of Diabetology and Endocrinology, Baqai Medical University, Karachi, PAK

**Keywords:** diabetes mellitis, type i diabetes mellitus, adolescent, children, linear growth

## Abstract

Objective

In this study, we aimed to determine the linear growth and final height in children/adolescents with type 1 diabetes mellitus (T1DM) at a tertiary care hospital.

Methodology

This observational study was conducted at the Baqai Institute of Diabetology and Endocrinology (BIDE), Baqai Medical University, Karachi, Pakistan. All children/adolescents diagnosed with T1DM of either gender aged between 8-18 years visiting the outpatient department of BIDE were included after obtaining informed consent. A predesigned questionnaire was developed to record data. The Centers for Disease Control and Prevention (CDC) growth chart was plotted and growth velocity was checked every six months to observe the linear growth. The final height was compared with the targeted height of the respective participants.

Results

A total of 66 people participated in the study (24 males and 42 females); among them, the mean age at diagnosis was 11.17 ± 4.77 years, and the duration of diabetes [median (IQR)] at the first visit was one year (0-3). The mean age at menarche was noted to be 13.56 ± 1.41 years. The overall height [standard deviation score (SDS)] at the first visit was -0.62 ± 2.58 and it was -1.34 ± 0.94 at the last visit; the overall weight at the first visit (SDS) and at the last visit was -1.23 ± 2.77 and -1.14 ± 1.25 respectively. Furthermore, the overall mid-parental height was 160.9 5 ± 10.28 cm, and 50% of males and 85.7% of females achieved genetic target height with a significant difference between them (p = 0.002).

Conclusion

A large number of people with TIDM were not able to achieve their target height. Therefore, it is imperative to monitor metabolic control along with monitoring of growth in young people with T1DM.

## Introduction

Type 1 diabetes mellitus (T1DM) is one of the most common chronic endocrine disorders among children and adolescents [1]. According to the International Diabetes Federation Atlas 9th edition 2019 estimates, around 1.98 billion children (0-14 years) and 2.58 billion adolescents (0-19 years) have type 1 diabetes globally [2]. In Pakistan, the incidence of T1DM has been reported to be 1.02 per 100,000 per year [3]. As the incidence of this disease is increasing worldwide, diabetic complications have come to represent a major concern despite the advances in treatment.

Impaired growth, one of the long-term consequences of T1DM, is described as a growth rate that is under the appropriate growth velocity for both age and gender [4]. Chronic hyperglycemia and severe insulin deficiency in people with T1DM are known to be associated with impaired linear growth, which is a complex physiological process influenced by nutritional, endocrinological, and psychological factors [5]. In children with uncontrolled T1DM, lower levels of growth hormones (GH), insulin-like growth factor (IGF) types I and II, their receptors, and high-affinity binding proteins such as IGF-binding proteins (IGFBP-1 to IGFBP-6) may also be observed. However, with the enhancement of glycemic control, the IGF type I level increases to produce a compensatory acceleration of growth [6]. It has been established that in people with T1DM, particularly during puberty, some alterations in the GH/IGF-1 with poor metabolic control exist, characterized by the hypersecretion of GH and a reduction in IGF-1 as well as an increase in IGFPB-1 levels [7]. It has also been reported that low IGF-1 levels cause reduced negative feedback to the pituitary gland and produce GH hypersecretion, the most important factor contributing to insulin resistance during pubertal age in people with T1DM [8]. However, previous studies have also reported that the decreased height growth may be determined by the duration of the disease rather than the degree of metabolic control [9].

In literature, the effects of T1DM on growth are still unclear and being debated. Data regarding linear growth is scarce in Pakistan. Therefore, this study was designed to determine the linear growth and final height in people with T1DM at a tertiary care hospital in Karachi, Pakistan.

## Materials and methods

This observational study was conducted at the Baqai Institute of Diabetology and Endocrinology (BIDE), a tertiary care center in Karachi, Pakistan. The duration of the study was 20 years, from January 2001 to April 2021. Ethical approval for the study was obtained from the Institutional review board at BIDE (IRB no. Ref: BIDE/IRB/WASHAIKH/08/20/20/0233). Informed consent was obtained from each participant prior to their enrolment in the study.

All children/adolescents diagnosed with T1DM of either gender aged between 8-18 years visiting the outpatient department of BIDE were included by using non-probability consecutive sampling technique, whereas people with a known case of celiac disease, thyroid disorders, and hypercortisolism were excluded. A predesigned questionnaire was developed for data records. It included demographic and anthropometric details, comprehensive history, clinical examination, treatment details, and relevant investigations.

The height of the study participants and parents was measured in centimeters (cm) using a stadiometer and mid-parental height was calculated by using the following formula:

In boys, [(Father's Height + Mother's Height + 13)/2], and in girls, [(Father's Height - 13 + Mother's Height)/2].

Weight was measured in kilograms (kg) on a calibrated digital weighing machine. The Centers for Disease Control and Prevention (CDC) growth chart was plotted and growth velocity was checked every six months to observe the linear growth. The final height was compared with the targeted height of the respective participants.

Statistical analysis

Summary statistics were expressed as arithmetic mean ± SD, median (IQR), or number (percentage). Analysis of variables was done using the Student's t-test, Mann-Whitney U test, chi-squared test, and Pearson correlation coefficient as appropriate. A p-value <0.05 was considered to be statistically significant.

## Results

Overall, 66 children/adolescents with type 1 diabetes (42 females and 24 males) were included in our study. The mean age at diagnosis was 12.92 ± 4.23 years in males and 10.17 ± 4.82 years in females, whereas the duration of diabetes at first visit was 0.5 years (IQR: 0-2.75) in males and one year (IQR: 0-3) in females (p = 0.733). The demographic characteristics and clinical parameters of the cohort are shown in Table 1.

**Table 1:** Demographic and clinical characteristics of the study participants (n = 66) P-value <0.05 considered statistically significant SD: standard deviation; SDS: standard deviation score; IQR: interquartile range

Variables	Male (n = 24)	Female (n = 42)	P-value	Overall (n = 66)
Age at diagnosis, years, mean ± SD	12.92 ± 4.23	10.17 ± 4.82	0.023	11.17 ± 4.77
HbA1c at first visit, %, mean ± SD	11.29 ± 3.48	11.07 ± 2.45	0.778	11.14 ± 2.81
HbA1c at last visit, %, mean ± SD	9.9 ± 3.03	9.02 ± 1.87	0.16	9.32 ± 2.33
Duration of diabetes at first visit, years, median (IQR)	0.5 (0-2.75)	1 (0-3)	0.733	1 (0-3)
Weight at first visit, SDS, mean ± SD	-1.81 ± 1.27	-0.9 ± 3.31	0.201	-1.23 ± 2.77
Weight at last visit, SDS, mean ± SD	-1.74 ± 1.14	-0.81 ± 1.2	0.003	-1.14 ± 1.25
Height at first visit, SDS, mean ± SD	-1.15 ± 1.01	-0.32 ± 3.12	0.211	-0.62 ± 2.58
Height at last visit, SDS, mean ± SD	-1.56 ± 1.01	-1.22 ± 0.89	0.165	-1.34 ± 0.94
BMI at first visit, SDS, mean ± SD	-1.75 ± 1.57	-1.12 ± 2	0.19	-1.35 ± 1.87
BMI at last visit, SDS, mean ± SD	-1.05 ± 1.42	-0.15 ± 1.18	0.007	-0.47 ± 1.34
Family history of diabetes, n (%)	9 (37.5%)	14 (33.3%)	0.733	23 (34.8%)

The comparison of targeted height in type 1 diabetes with respect to gender revealed that 12 (50%) males and most of the females (36, 85.7%) achieved their targeted height with a significant difference between them (p = 0.002), as shown in Table 2.

**Table 2:** Genderwise comparison of targeted height achievement in study participants

Targeted height	Male	Female	P-value	Overall
N	24	42		66
Not achieved	12 (50%)	6 (14.3%)	0.002	18 (27.3%)
Achieved	12 (50%)	36 (85.7%)		48 (72.7%)

**Figure 1:**
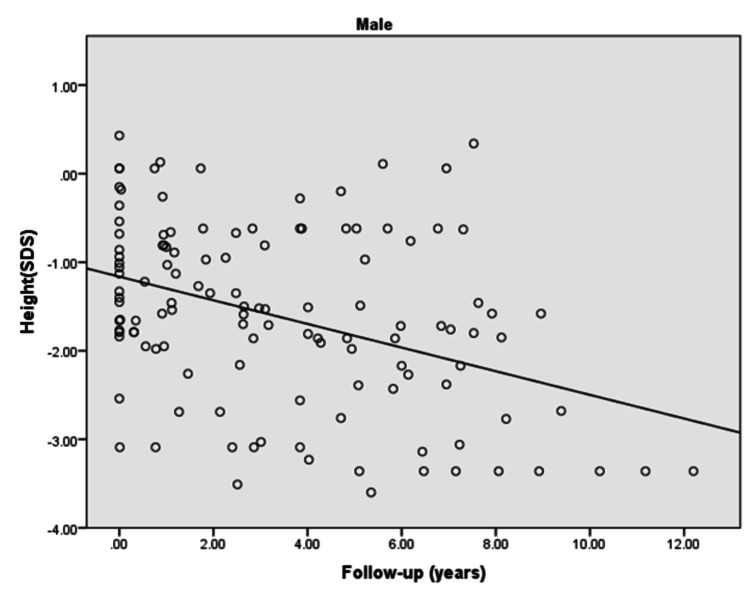
Height (SDS) of diabetic male children and adolescents in the follow-up duration r = -0.382; p<0.0001 SDS: standard deviation score

**Figure 2:**
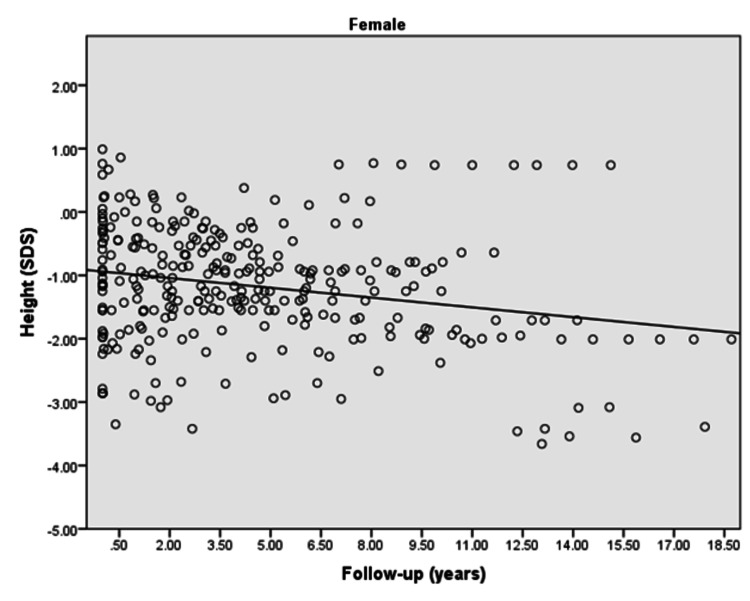
Height (SDS) of diabetic female children and adolescents in the follow-up duration r = -0.231; p<0.0001 SDS: standard deviation score

The height [standard deviation score (SDS)] of males and females with type 1 diabetes significantly decreased with the increased duration of diabetes (p: <0.0001). Furthermore, there was a negative correlation observed between height and duration of diabetes in males (r = -0.382) and females (r = -0.231) during the follow-up as revealed in Figures 1, 2.

**Figure 3:**
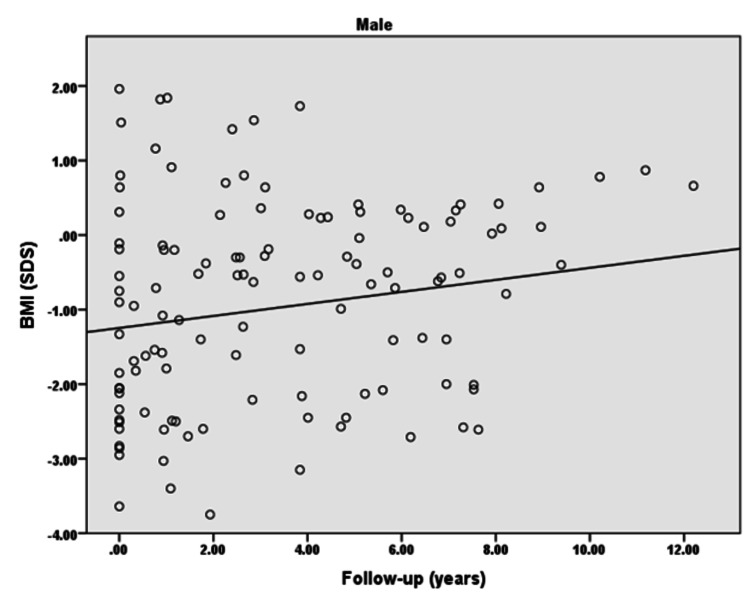
BMI (SDS) of diabetic male children and adolescents in the follow-up duration r = 0.163; p = 0.07 BMI: body mass index; SDS: standard deviation score

**Figure 4:**
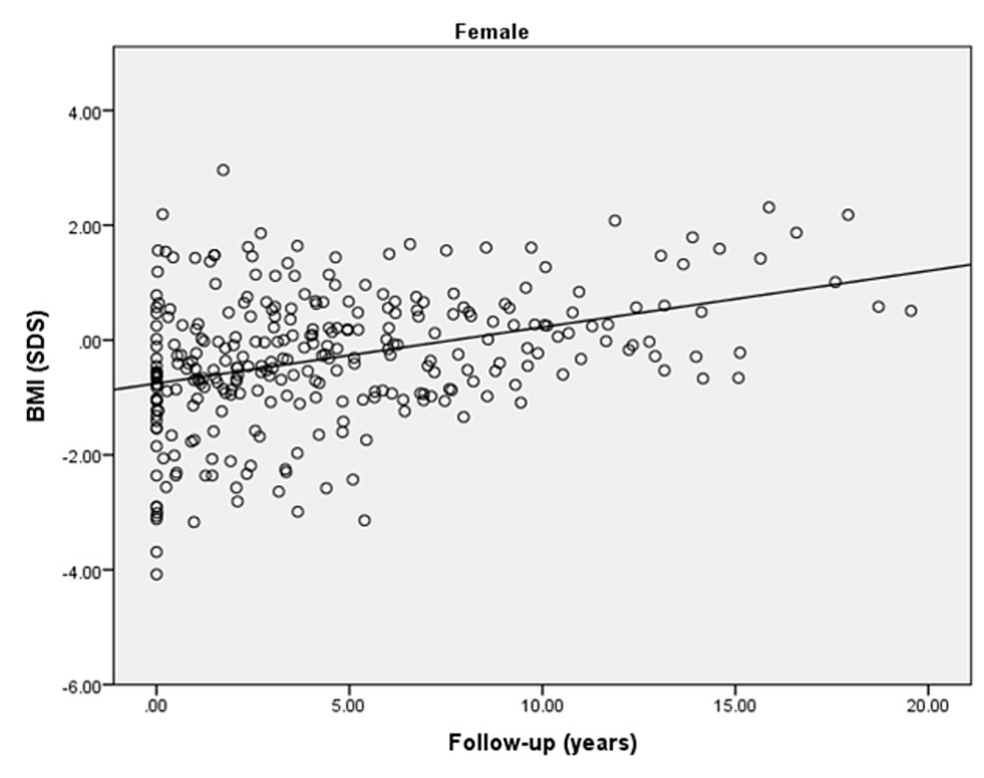
BMI (SDS) of diabetic female children and adolescents in the follow-up duration r = 0.358; p<0.0001 BMI: body mass index; SDS: standard deviation score

The BMI (SDS) of males with type 1 diabetes revealed that BMI insignificantly increased with the increased duration of diabetes (p = 0.07). Furthermore, there was a weak positive correlation observed between BMI and the duration of diabetes in males (r = 0.163), as shown in Figure 3. However, female children/adolescents with type 1 diabetes had a significant increase in their BMI (SDS) with the increasing duration of diabetes (p: <0.0001). Moreover, there was a moderate positive correlation observed between BMI and the duration of diabetes in females in the follow-up duration (r = 0.358), as shown in Figure 4.

## Discussion

The present study demonstrated that the majority of the females with type 1 diabetes achieved targeted height whereas only half of the males reached the targeted height. Moreover, a significantly negative and weak correlation was found for height (SDS) of children/adolescents with the duration of diabetes in both males and females in the follow-up duration. Furthermore, a significantly positive weak correlation for BMI (SDS) with the duration of diabetes was observed in females in the follow-up duration.

In our study, the mean age at diagnosis of diabetes was 11.17 years. The mean height (SDS) for boys and girls at the first visit was -1.15 and -0.32 respectively, whereas the mean final height (SDS) at the last visit for boys and girls was -1.56 and -1.22 respectively. In contrast to our findings, another longitudinal study showed a mean age of 8.70 years at the beginning of the disease and reported the mean final height (SDS) for boys as +0.14 and that for girls as -0.57. The final heights of girls in that study were considerably shorter as compared to their targeted heights [10]. In this study, the mean height of males was -1.15 ± 1.01 and it was -0.32 ± 3.12 in females at the time of presentation with an insignificant difference between them. In contrast with this, the research done by Bonfig et al. who studied 22,651 Austrian and German children with T1DM revealed that their mean height (SDS) at the time of diagnosis was substantially greater than that of the normal healthy people [11].

Various data analyses have shown that children with T1DM demonstrate a decrease in height (SDS) and growth rate following the onset of the disease in the prepubertal years [12]. Moreover, the growth velocity is likely to be affected by the age of onset of T1DM, with the most severe impairment of growth occurring in children with disease onset at early childhood [11-13]. Our study findings are consistent with those of the above-mentioned study and showed that children with T1DM showed decreased height (SDS) subsequent to disease onset in the prepubertal years.

Based on further interpretation, a lot of researchers have analyzed the growth pattern as a prognostic factor for the progression of disease in children prior to the onset of T1DM [14,15]. Several studies have verified that a quick gain of weight, height, and BMI in the prepubertal stage relates to the progression of islet autoimmunity and, subsequently, the beginning of T1DM in children [12,16]. However, in our study, growth parameters such as height, weight, and BMI (SDS) were lower than those of healthy peers. The height increased while weight and BMI significantly decreased with the increased duration of diabetes, but the height was still below that of the healthy peers. Another study has shown that after the achievement of final height in girls with type 1 diabetes, their mean weight-for-height (SDS) was +0.76, indicating that they have a propensity to grow obese [10]. Similarly, our study revealed that BMI significantly increased with the increased duration of diabetes predominantly in females while height (SDS) significantly decreased with the increased duration of diabetes. In spite of the described growth abnormalities, many studies have demonstrated that T1DM children attain a normal or only slightly decreased ultimate height than the mentioned population [17-19].

A few studies have described a relationship between the duration of T1DM and final adult height, with poorer growth in patients with a prolonged period of diabetes [20,21]. Our study findings are consistent with the above-reported study and showed that only half of the boys achieved targeted height, whereas most of the girls attained their targeted heights. Our results are also in agreement with an earlier study that demonstrated sex-specific differences in terms of these parameters. It was found that during the pubertal growth, girls reached the peak of height velocity due to higher serum IGF-I and estrogens levels in females having a stronger impact than androgens on GH signaling [22].

Another important factor that influences final height in T1DM patients is glycemic control [23,24]. A study by Bonfig et al. involving 1,685 German children with T1DM observed a mean final adult height of 167.1 ± 6.2 cm (SDS: -0.16 ± 0.97) in girls and 179.6 ± 7.1 cm (SDS: -0.17 ± 1) in boys with an insignificant difference between both genders; the studied children were further classified on the basis of glycemic control and they observed that groups with HbA1c <7% achieved an ultimate adult height (SDS) of +0.030, while groups with HbA1c levels of 7-8% had an ultimate adult height (SDS) of -0.122, and those with sub-standard metabolic control (HbA1c >8.0%) had an ultimate adult height (SDS) of -0.308 [25]. Our study is in agreement with the above-cited studies and reported the mean final height (SDS) to be -1.56 ± 1.01 in males and -1.22 ± 0.89 in females. Furthermore, the mean final HbA1c was found to be 9.9 ± 3.03% in males and 9.02 ± 1.87% in females, indicating that poorly controlled glycemic levels tended to reduce height irrespective of gender.

This is a unique study as data regarding linear growth in Pakistani adolescents with T1DM is limited. However, our study has a few limitations. This was a single-center analysis and the sample size was relatively small. Also, there were some confounders that may have affected the target height (i.e., socioeconomic status and nutrition), which were not taken into account.

## Conclusions

Based on our findings, a large number of people with type 1 diabetes were not able to achieve their targeted height. Therefore, it is crucial to monitor metabolic control along with the monitoring of growth in young people with type 1 diabetes to reduce the risk of growth impairment. Our results also revealed that weight and BMI significantly increased with poor glycemic control and the duration of diabetes.

## References

[1] Krzewska A, Ben-Skowronek I (2016). Effect of associated autoimmune diseases on type 1 diabetes mellitus incidence and metabolic control in children and adolescents. Biomed Res Int.

[2] (2022). IDF Diabetes Atlas. http://www.diabetesatlas.org.

[3] Ahmedani MY, Fawwad A, Shaheen F, Tahir B, Waris N, Basit A (2019). Optimized health care for subjects with type 1 diabetes in a resource constraint society: a three-year follow-up study from Pakistan. World J Diabetes.

[4] Santi E, Tascini G, Toni G, Berioli MG, Esposito S (2019). Linear growth in children and adolescents with type 1 diabetes mellitus. Int J Environ Res Public Health.

[5] Kim MS, Quintos JB (2008). Mauriac syndrome: growth failure and type 1 diabetes mellitus. Pediatr Endocrinol Rev.

[6] Kharagjitsingh AV, de Ridder MA, Roep BO, Koeleman BP, Bruining GJ, Veeze HJ (2010). Revisiting infant growth prior to childhood onset type 1 diabetes. Clin Endocrinol (Oxf).

[7] Raisingani M, Preneet B, Kohn B, Yakar S (2017). Skeletal growth and bone mineral acquisition in type 1 diabetic children; abnormalities of the GH/IGF-1 axis. Growth Horm IGF Res.

[8] Clark PA, Clarke WL, Pedadda S, Reiss A, Langlois C, Nieves-Rivera F, Rogol AD (1998). The effects of pubertal status and glycemic control on the growth hormone-IGF-I axis in boys with insulin-dependent diabetes mellitus. J Pediatr Endocrinol Metab.

[9] Aljuhani FM, Al-Agha AE, Almunami BA (2018). Growth status of children and adolescents with type 1 diabetes mellitus in Jeddah, Saudi Arabia: a cross-sectional study. Curr Pediatr Res.

[10] Wong GW, Cheng PS, Leung TF (2000). Sex differences in the growth of diabetic children. Diabetes Res Clin Pract.

[11] Bonfig W, Kapellen T, Dost A, Fritsch M, Rohrer T, Wolf J, Holl RW (2012). Growth in children and adolescents with type 1 diabetes. J Pediatr.

[12] Magnus MC, Olsen SF, Granström C (2015). Infant growth and risk of childhood-onset type 1 diabetes in children from 2 Scandinavian birth cohorts. JAMA Pediatr.

[13] Bizzarri C, Benevento D, Giannone G (2014). Sexual dimorphism in growth and insulin-like growth factor-I in children with type 1 diabetes mellitus. Growth Horm IGF Res.

[14] Elding Larsson H, Vehik K, Haller MJ (2016). Growth and risk for islet autoimmunity and progression to type 1 diabetes in early childhood: The Environmental Determinants of Diabetes in the Young Study. Diabetes.

[15] Yassouridis C, Leisch F, Winkler C, Ziegler AG, Beyerlein A (2017). Associations of growth patterns and islet autoimmunity in children with increased risk for type 1 diabetes: a functional analysis approach. Pediatr Diabetes.

[16] Giannini C, Mohn A, Chiarelli F (2014). Growth abnormalities in children with type 1 diabetes, juvenile chronic arthritis, and asthma. Int J Endocrinol.

[17] Chiarelli F, Giannini C, Mohn A (2004). Growth, growth factors and diabetes. Eur J Endocrinol.

[18] Bizzarri C, Timpanaro TA, Matteoli MC, Patera IP, Cappa M, Cianfarani S (2018). Growth Trajectory in Children with Type 1 Diabetes Mellitus: The Impact of Insulin Treatment and Metabolic Control. Horm Res Paediatr.

[19] Luna R, Alvarez-Vázquez P, Hervás E, Casterás A, Pérez Méndez L, Páramo C, García-Mayor RV (2005). The role of diabetes duration, pubertal development and metabolic control in growth in children with type 1 diabetes mellitus. J Pediatr Endocrinol Metab.

[20] Elamin A, Hussein O, Tuvemo T (2006). Growth, puberty, and final height in children with type 1 diabetes. J Diabetes Complications.

[21] Parthasarathy L, Khadilkar V, Chiplonkar S, Khadilkar A (2016). Longitudinal growth in children and adolescents with type 1 diabetes. Indian Pediatr.

[22] Cole TJ, Ahmed ML, Preece MA, Hindmarsh P, Dunger DB (2015). The relationship between insulin-like growth factor 1, sex steroids and timing of the pubertal growth spurt. Clin Endocrinol (Oxf).

[23] Demir K, Altıncık A, Abacı A, Büyükgebiz A, Böber E (2010). Growth of children with type 1 diabetes mellitus. J Clin Res Pediatr Endocrinol.

[24] Ekström K, Salemyr J, Zachrisson I, Carlsson-Skwirut C, Ortqvist E, Bang P (2007). Normalization of the IGF-IGFBP axis by sustained nightly insulinization in type 1 diabetes. Diabetes Care.

[25] Sami W, Ansari T, Butt NS, Hamid MR (2017). Effect of diet on type 2 diabetes mellitus: a review. Int J Health Sci (Qassim).

